# Macrophages Provide Essential Support for Erythropoiesis, and Extracellular ATP Contributes to a Erythropoiesis-Supportive Microenvironment during Repeated Psychological Stress

**DOI:** 10.3390/ijms241411373

**Published:** 2023-07-12

**Authors:** Sanja Momčilović, Andrija Bogdanović, Maja S. Milošević, Slavko Mojsilović, Dragana C. Marković, Dušica M. Kočović, Sanja Vignjević Petrinović

**Affiliations:** 1 Group for Neuroendocrinology, Institute for Medical Research, National Institute of Republic of Serbia, University of Belgrade, 11129 Belgrade, Serbia; sanja.momcilovic@imi.bg.ac.rs (S.M.); mmilosevic@imi.bg.ac.rs (M.S.M.); dusica.kocovic@imi.bg.ac.rs (D.M.K.); 2 Clinic for Hematology, Clinical Center of Serbia, Faculty of Medicine, University of Belgrade, 11129 Belgrade, Serbia; ebogdano@eunet.rs; 3 Group for Hematology and Stem Cells, Institute for Medical Research, National Institute of Republic of Serbia, University of Belgrade, 11129 Belgrade, Serbia; slavko@imi.bg.ac.rs; 4 Group for Immunology, Institute for Medical Research, National Institute of Republic of Serbia, University of Belgrade, 11129 Belgrade, Serbia; dragana.markovic@imi.bg.ac.rs

**Keywords:** macrophages, stress, erythropoiesis, extracellular ATP, microenvironment

## Abstract

Psychological stress is a significant contributor to various chronic diseases and affects multiple physiological processes including erythropoiesis. This study aimed to examine the tissue-specific contributions of macrophages and extracellular ATP, as a signal of disturbed tissue homeostasis, to erythropoiesis under conditions of repeated psychological stress. Adult male BALB/c mice were subjected to 2 h daily restraint stress for seven consecutive days. Clodronate-liposomes were used to deplete resident macrophages from the bone marrow and spleen two days prior to the first restraint procedure, as well as newly recruited macrophages, every third day for the duration of the experiment. Repeated stress induced a considerable increase in the number of erythroid progenitor cells as well as in the percentage of CD71+/Ter119+ and CD71−/Ter119+ cells in the bone marrow and spleen. Macrophage depletion completely abolished the stimulative effect of repeated stress on immature erythroid cells, and prevented stress-induced increases in ATP levels, P2X7 receptor (P2X7R) expression, and ectonucleotidase CD39 activity and expression in the bone marrow and spleen. The obtained results demonstrate the stimulative effects of repeated stress on erythroid cells, extracellular ATP levels, P2X7R expression, CD39 activity and expression within the bone marrow and spleen, as well as the essential role of macrophages in stress-induced changes.

## 1. Introduction

Erythropoiesis plays a critical role in maintaining oxygen delivery to the tissues under both physiological and pathological conditions. Unlike steady-state erythropoiesis, which refers to a continuous process of red blood cell (RBC) formation under homeostatic conditions, stress erythropoiesis implies a rapid production of erythrocytes during times of considerable erythropoietic need [1]. Stress erythropoiesis occurs in response to anemia due to severe blood loss, hypoxia, stress, inflammation, etc. [2,3,4]. The signals that drive stress erythropoiesis differ greatly from those regulating homeostatic RBC production. The mechanisms of accelerated RBC production are strongly dependent on niche-derived signals, such as bone morphogenic protein 4 (BMP4) [5,6,7]. Stress erythropoiesis is a highly conserved pathway in mice and humans and utilizes a distinct self-renewing progenitor population, also known as “stress” erythroid progenitors [8]. The erythroid progenitors are located in proximity to macrophages, which provide signals that affect erythroid cell proliferation and differentiation [9].

Apart from well-known immune functions, macrophages play additional roles including the regulation of the hematopoietic microenvironment [10,11]. Thus, bone marrow macrophages have emerged as important regulators of hematopoietic stem and progenitor cell (HSPC) mobilization [12]. Furthermore, the interactions between macrophages and immature erythroid cells occur within the specialized niches in the bone marrow and spleen [13]. The spleen contains a favorable microenvironment for an extensive expansion of erythroid progenitors in response to stressful stimuli [7,14,15]. Accumulating data point towards the dynamic nature of the spleen and show that monocyte-derived macrophages expand the erythropoietic niche in response to anemic stress [16]. Considering macrophage motility is as an important aspect of their function, macrophages are poised to rapidly respond to diverse migratory stimuli from the tissue microenvironment [17].

Extracellular nucleotides have been implicated in controlling the movement of macrophages [18]. Adenosine triphosphate (ATP) is one of the endogenous danger signals that macrophages can detect in the surrounding microenvironment [19]. ATP is released from cells as a result of disturbed tissue homeostasis in response to tissue damage, stress, or inflammation. Macrophages migrate towards increasing ATP concentrations and possess P2X7 receptors (P2X7R), which respond to high ATP levels, as well as the ectonucleotidase CD39 that rapidly converts ATP to adenosine monophosphate [18,20].

Using a murine model of psychological stress, we have previously shown that chronic stress stimulates erythropoiesis both in the bone marrow and spleen [7,21,22,23]. In this study, we extended our previous research, and examined tissue-specific contributions of macrophages and extracellular ATP to erythropoiesis under conditions of repeated psychological stress.

## 2. Results

### 2.1. The Effects of Macrophage Depletion on Hematological Parameters

The depletion of macrophages decreased the number of circulating RBCs, hematocrit and hemoglobin blood levels under both basal and repeated stress conditions (Table 1). The number of RBCs and hemoglobin levels were significantly lower in mice treated with clodronate-liposome and subjected to repeated stress, compared with repeatedly stressed mice.

### 2.2. Depletion of Macrophages Abolishes the Effects of Repeated Restraint Stress on Erythroid Progenitors in the Bone Marrow and Spleen

**Figure 1:**
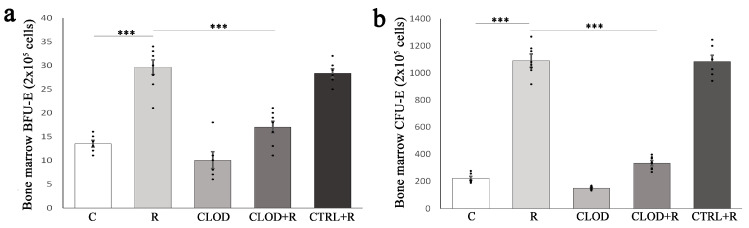
Effects of repeated restraint stress and macrophage depletion on (**a**) BFU-E and (**b**) CFU-E in the bone marrow. C—control (untreated animals), R—animals subjected to daily restraint stress for 7 consecutive days, CLOD—animals treated with clodronate liposomes, CLOD + R—animals treated with clodronate liposomes and subjected to daily restraint stress, CTRL + R—animals received control liposomes and subjected to daily restraint stress. Data are expressed as mean ± SEM (*n* = 6–8/group) *** *p* < 0.001 (one-way ANOVA following Games–Howell and Bonferroni-corrected *t* test as appropriate).

**Figure 2:**
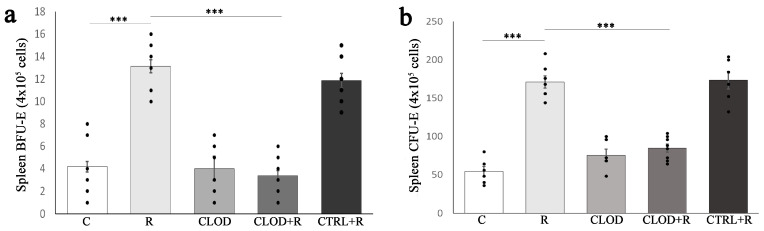
The effects of repeated restraint stress and macrophage depletion on (**a**) BFU-E and (**b**) CFU-E in the spleen. C—control (untreated animals), R—animals subjected to daily restraint stress for 7 consecutive days, CLOD—animals treated with clodronate-liposomes, CLOD + R—animals treated with clodronate-liposomes and subjected to daily restraint stress, CTRL + R—animals received control liposomes and subjected to daily restraint stress. Data are expressed as mean ± SEM (*n* = 6–8/group) *** *p* < 0.001 (one-way ANOVA following Games–Howell and Bonferroni-corrected *t* test as appropriate).

In order to examine the contribution of macrophages to erythropoiesis under conditions of repeated psychological stress, we induced macrophage depletion with clodronate-liposomes, an effective method commonly utilized to investigate the role of macrophages in vivo. Clodronate-encapsulated liposomes target macrophage uptake by phagocytosis and induce macrophage apoptosis after phagocytosing the liposomes. Intraperitoneally injected clodronate-liposomes were used to deplete resident macrophages from the bone marrow and spleen two days prior to the first restraint procedure, as well as newly recruited macrophages, every third day for the duration of experiment. We used the Western blot technique and showed a notable decrease in F4/80 protein expression in the bone marrow and spleen of clodronate-liposome-treated mice (Supplement Figure S1). Initially, we examined the effects of macrophage depletion on the earliest committed erythroid progenitors, including burst-forming unit-erythroid (BFU-E) and colony forming unit-erythroid (CFU-E), in the bone marrow and spleen. Repeated exposure to restraint stress induced a great increase in the number of BFU-E and CFU-E cells in the bone marrow (Figure 1) and spleen (Figure 2).

Depletion of macrophages did not significantly alter the number of erythroid progenitors under basal conditions but completely prevented the effect of repeated stress on BFU-E (Figure 1a and Figure 2a) and CFU-E (Figure 1b and Figure 2b) cells in both the bone marrow and spleen.

### 2.3. The Effects of Macrophage Depletion on Ter119-Positive Cells in the Bone Marrow and Spleen under Conditions of Repeated Stress

**Figure 3:**
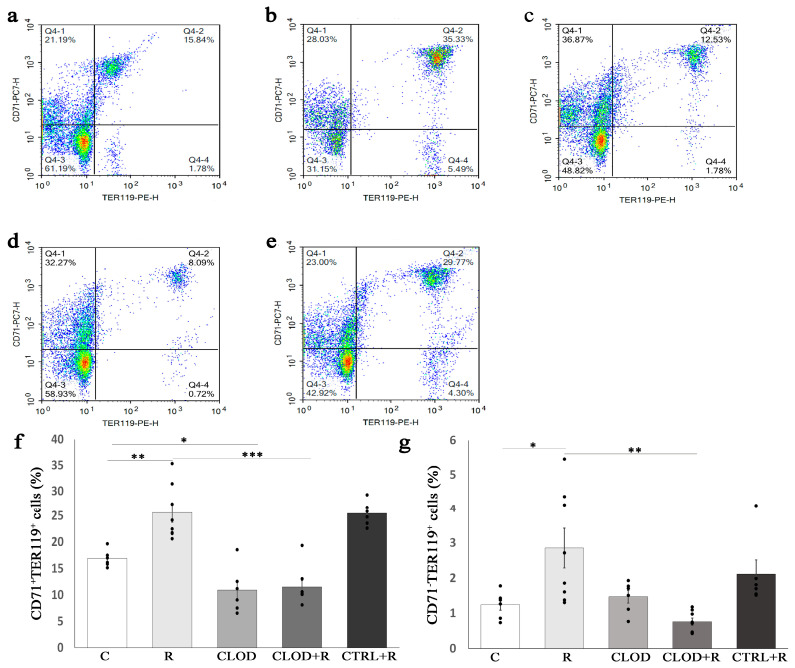
Effects of repeated restraint stress and macrophage depletion on erythroid precursors in the bone marrow: (**a**) representative dot plots of erythroid precursors at different maturational stages in the bone marrow of control mice, (**b**) animals subjected to daily restraint stress for 7 consecutive days, (**c**) animals treated with clodronate-liposomes, (**d**) animals treated with clodronate-liposomes and subjected to daily restraint, and (**e**) animals received control liposomes and subjected to daily restraint. The main erythroid cell populations, (**f**) CD71+/Ter119+ and (**g**) CD71−/Ter119+, are presented as a percentage of all bone marrow cells. Data are expressed as mean ± SEM (*n* = 6–8/group). * *p* < 0.05, ** *p* < 0.01, *** *p* < 0.001 (one-way ANOVA following Games–Howell and Fisher’s least significant difference test as appropriate).

**Figure 4:**
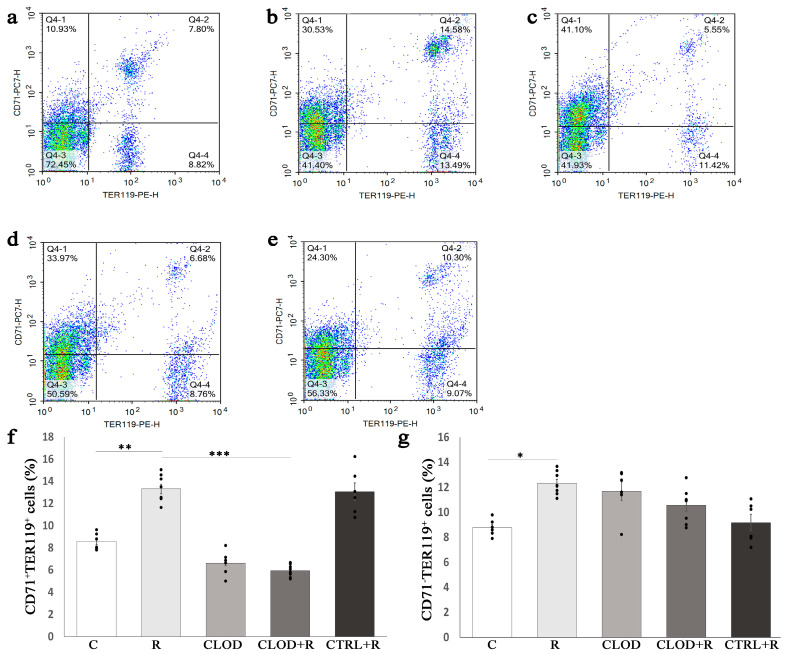
Effects of repeated restraint stress and macrophage depletion on erythroid precursors in the spleen: (**a**) representative dot plots of erythroid precursors at different maturational stages in the bone marrow of control mice, (**b**) animals subjected to daily restraint stress for 7 consecutive days, (**c**) animals treated with clodronate-liposomes, (**d**) animals treated with clodronate-liposomes and subjected to daily restraint, and (**e**) animals received control liposomes and subjected to daily restraint. The main erythroid cell populations, (**f**) CD71+/Ter119+ and (**g**) CD71−/Ter119+, are presented as a percentage of all splenocytes. Data are expressed as mean ± SEM (*n* = 6–8/group). * *p* < 0.05, ** *p* < 0.01, *** *p* < 0.001 (one-way ANOVA following Games–Howell and Bonferroni-corrected *t* test as appropriate).

Ter119 antigen is highly expressed on erythroid cells from pro-erythroblast through mature erythrocyte stages, and we next analyzed the effects of macrophage depletion on the percentage of Ter119+ cells upon repeated stress. As transferrin receptor (CD71) is also expressed on the surface of erythroid cells, and the vast majority of mature erythrocytes lack CD71 expression, we used flow cytometry to analyze the CD71/Ter119 profile of bone marrow and spleen cells following macrophage depletion and/or repeated exposure to daily restraint stress (Supplement Figure S2). Repeated stress exposure resulted in a significantly increased percentage of CD71+/Ter119+ and CD71−/Ter119+ in the bone marrow (Figure 3) and spleen (Figure 4).

Treatment with clodronate-liposomes abrogated the effect of stress on CD71+/Ter119+ (Figure 3f and Figure 4f) in the bone marrow and spleen, as well as on CD71−/Ter119+ cells in the bone marrow (Figure 3g). Macrophage depletion under basal conditions reduced the percentage of CD71+/Ter119+ cells (Figure 3f), but CD71−/Ter119+ cells (Figure 3g) remained unchanged in the bone marrow. In contrast to bone marrow, treatment with clodronate-liposomes under steady-state conditions did not significantly alter the percentage of CD71+/Ter119+ cells in the spleen (Figure 4f).

### 2.4. The Effects of Macrophage Depletion on Different Subpopulations of Ter119-Positive Cells in the Bone Marrow and Spleen upon Repeated Stress

**Figure 5:**
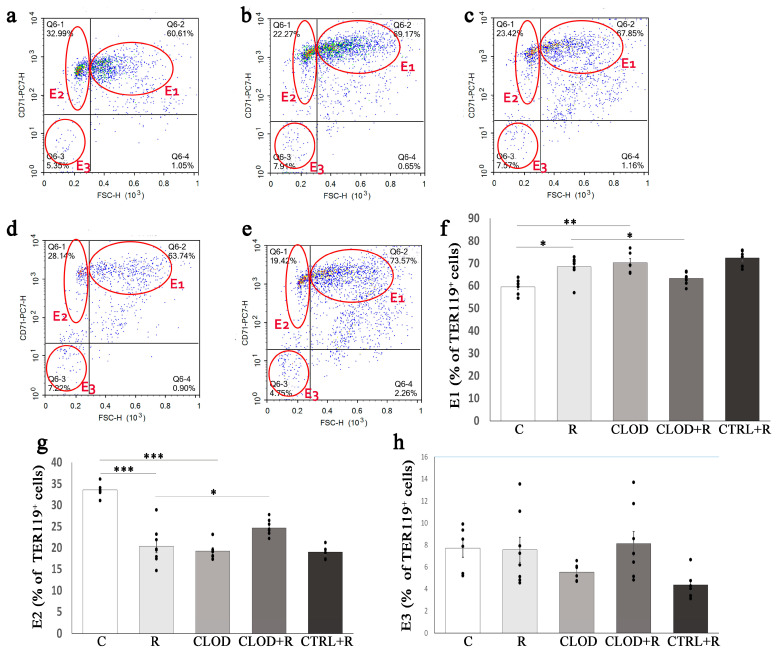
Changes in the proportion of bone marrow Ter119+ cell subpopulations during repeated restraint stress and macrophage depletion. The total Ter119+ bone marrow cells were analyzed for their size (forward-scatter parameter) and CD71 expression, and accordingly divided into three increasingly mature cell subpopulations (E1, E2 and E3). (**a**) Representative dot plots of Ter119+ cell subpopulations in the bone marrow of control mice; (**b**) animals subjected to daily restraint stress for 7 consecutive days; (**c**) animals treated with clodronate-liposomes; (**d**) animals treated with clodronate-liposomes and subjected to daily restraint; and (**e**) animals received control liposomes and subjected to daily restraint. Percentage participation of (**f**) E1 subpopulation, (**g**) E2 subpopulation, and (**h**) E3 subpopulation of bone marrow Ter119+ cell subpopulations. Data are expressed as mean ± SEM (*n* = 6–8/group). * *p* < 0.05, ** *p* < 0.01, *** *p* < 0.001 (one-way ANOVA following Bonferroni-corrected *t* test as appropriate).

**Figure 6:**
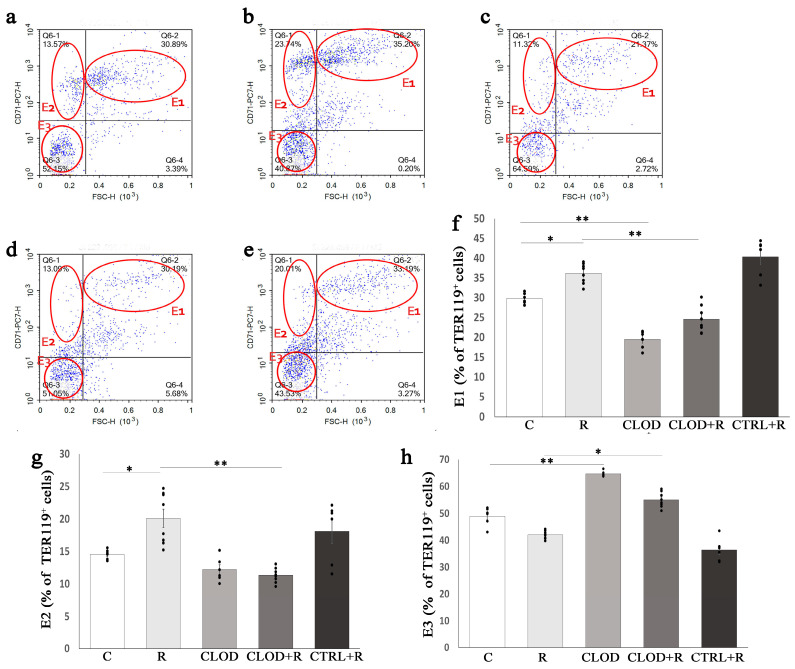
Changes in the proportion of spleen Ter119+ cell subpopulations during repeated restraint stress and macrophage depletion. The total Ter119+ splenocytes were analyzed for their size (forward-scatter parameter) and CD71 expression, and accordingly divided into three increasingly mature cell subpopulations (E1, E2 and E3). (**a**) Representative dot plots of Ter119+ cell subpopulations in the bone marrow of control mice, (**b**) animals subjected to daily restraint stress for 7 consecutive days, (**c**) animals treated with clodronate-liposomes, (**d**) animals treated with clodronate-liposomes and subjected to daily restraint, (**e**) animals received control liposomes and subjected to daily restraint, (**f**) percentage participation of E1 subpopulation, (**g**) E2 subpopulation, and (**h**) E3 subpopulation of Ter119+ splenocytes. Data are expressed as mean ± SEM (*n* = 6–8/group). * *p* < 0.05, ** *p* < 0.01 (one-way ANOVA following Games–Howell as appropriate).

We further analyzed the size of Ter119+ cells, classified these cells into three subsets according to their maturation stage (E1, E2, E3), and examined the effects of clodronate-liposome treatment on subpopulations of Ter119+ cells in the bone marrow (Figure 5) and spleen (Figure 6) of repeatedly stressed mice. Daily exposure to restraint stress increased the percentage of most immature subpopulations of Ter119+ cells (E1) in both the bone marrow (Figure 5f) and spleen (Figure 6f), and macrophage depletion prevented this stress-induced increase. Treatment with clodronate-liposome under basal conditions significantly increased the percentage of the E1 subpopulation in the bone marrow (Figure 5f), whereas this treatment considerably reduced their percentage in the spleen (Figure 6f). The percentage of E2 Ter119+ cells was substantially decreased in the bone marrow (Figure 5g), but significantly increased (Figure 6g) in the spleen of repeatedly stressed mice. Depletion of macrophages countered these effects of stress on the E2 subpopulation of Ter119+ cells (Figure 5g and Figure 6g). Clodronate-liposome treatment under steady-state conditions markedly reduced the percentage of E2 Ter119+ cells in the bone marrow, whilst the number of these cells in the spleen remain unchanged. The proportion of the most mature subpopulation of Ter119+ cells (E3) in the bone marrow was not significantly altered following either repeated stress or macrophage depletion (Figure 5h). Unlike in the bone marrow, treatment with clodronate-liposomes significantly increased the percentage of E3 Ter119+ cells in the spleen under both basal and stress conditions (Figure 6h).

### 2.5. Repeated Restraint Stress Increases ATP Levels and P2X7R Expression in the Bone Marrow and Spleen

**Figure 7:**
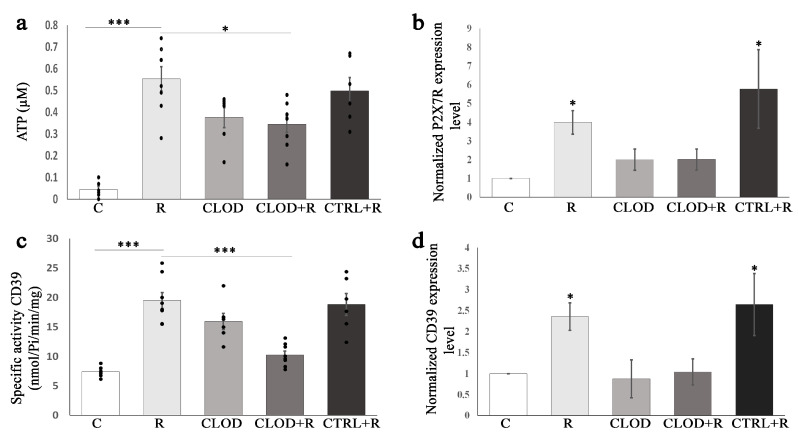
Effects of repeated restraint stress and macrophage depletion on (**a**) extracellular ATP levels, and (**c**) specific activity CD39 in the bone marrow. Data are expressed as mean ± SEM (*n* = 6–8/group). * *p* < 0.05, *** *p* < 0.001 (one-way ANOVA following Bonferroni-corrected as appropriate). (**b**) P2X7R mRNA and (**d**) CD39 mRNA expression changes under flow of repeated restraint stress and macrophage depletion in the bone marrow. Relative expression of P2X7R mRNA and CD39 mRNA were normalized to β actin and reported as fold change compared with untreated controls. Data are expressed as mean ± SEM (*n* = 6–8/group). Differences between groups were determined by Relative Expression Software Tool (REST, https://www.gene-quantification.de/rest-2009.html) 2009 using the pairwise fixed randomization test. * *p* < 0.05. C—controls (untreated animals), R—animals subjected to daily restraint stress for 7 consecutive days, CLOD—animals treated with clodronate-liposomes, CLOD + R—animals treated with clodronate-liposomes and subjected to daily restraint, CTRL + R—animals received control liposomes and subjected to daily restraint.

**Figure 8:**
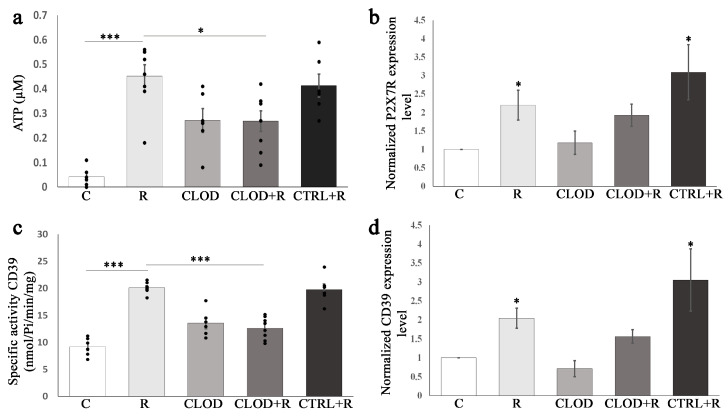
Effects of repeated restraint stress and macrophage depletion on (**a**) extracellular ATP levels, and (**c**) specific activity CD39 in the spleen. Data are expressed as mean ± SEM (*n* = 6–8/group). * *p* < 0.05, *** *p* < 0.001 (one-way ANOVA following Bonferroni-corrected as appropriate). (**b**) P2X7R mRNA and (**d**) CD39 mRNA expression changes under flow of repeated restraint stress and macrophage depletion in the spleen. Relative expression of P2X7R mRNA and CD39 mRNA were normalized to β actin, and reported as fold change compared with untreated controls. Data are expressed as mean ± SEM (*n* = 6–8/group). Differences between groups were determined by Relative Expression Software Tool (REST, https://www.gene-quantification.de/rest-2009.html) 2009 using the pairwise fixed randomization test. * *p* < 0.05. C—controls (untreated animals), R—animals subjected to daily restraint stress for 7 consecutive days, CLOD—animals treated with clodronate-liposomes, CLOD + R—animals treated with clodronate-liposomes and subjected to daily restraint, CTRL + R—animals received control liposomes and subjected to daily restraint.

Extracellular ATP, released from cells through exocytosis, binds to ATP-gated ion channel receptor P2X7, which is widely expressed in hematopoietic stem/progenitor cells and their lineages, including in macrophages. We next examined the effects of repeated stress on ATP levels and P2X7R expression in the bone marrow (Figure 7) and spleen (Figure 8). Repeated exposure to daily stress markedly increased the extracellular ATP levels in both the bone marrow (Figure 7a) and spleen (Figure 8a). Macrophage depletion reduced this stress-induced increase in bone marrow and splenic extracellular ATP concentrations. Upon repeated restraint stress, the expression levels of the P2X7R gene were significantly enhanced in the bone marrow (Figure 7b) and spleen (Figure 8b). Clodronate treatment did not change the expression of P2X7R gene under basal conditions, whereas it prevented stress-induced increase in P2X7R expression.

### 2.6. Enhanced Specific Activity and Expression Levels of the Ectonucleotidase CD39 in the Bone Marrow and Spleen during Repeated Stress

Once released in extracellular space, ATP is rapidly hydrolyzed by CD39 to maintain its physiological levels and prevent extracellular ATP-induced pathological effects. Therefore, we also analyzed the effects of repeated stress on the specific activity and the expression of CD39 in the bone marrow (Figure 7) and spleen (Figure 8). The increased activity of CD39 was detected in both the bone marrow (Figure 7c) and spleen (Figure 8c) of repeatedly stressed mice, whereas macrophage depletion abolished this effect of stress. The expression of CD39 was significantly upregulated in the bone marrow (Figure 7d) and spleen (Figure 8d) of repeatedly stressed mice. Treatment with clodronate-liposomes did not alter CD39 expression levels under basal conditions but prevented stress-induced elevation of CD39 expression in the bone marrow and spleen.

## 3. Discussion

Psychological stress is gaining increasing attention as a significant contributor to various chronic diseases, such as cardiovascular disease, diabetes, and cancer [24,25,26]. Repeated stress has profound effects on the immune system and is considered as one of the major triggers for persistent inflammation [27,28]. In addition to the well-established effects of stress on leukocyte distribution and function, accumulating evidence now demonstrates that chronic stress affects erythropoiesis as well [2,29]. We have previously demonstrated that chronic exposure to restraint stress, which acts primarily as a psychological stressor [30,31], induces anemia and stimulates erythropoiesis in the bone marrow and spleen [7,22]. In this study, we extended our previous findings and showed that depletion of macrophages further worsened anemia and abolished the effects of repeated stress on immature erythroid cells in the bone marrow and spleen. We also found that repeated exposure to psychological stress increased ATP levels, purinergic P2X7R expression, and CD39 specific activity and expression in the bone marrow and spleen.

Macrophage depletion decreased the number of circulating RBCs, hematocrit and hemoglobin blood levels under both basal and repeated stress conditions. In accordance with our results, Bader et al. [32] have shown that clodronate treatment results in anemia by decreasing the absolute hemoglobin levels and the hematocrit in mice. Furthermore, decreased hemoglobin concentrations and RBC have been demonstrated in clodronate-treated mice 7 days following phlebotomy-induced anemia [33]. Our results also showed significantly lower RBC numbers and hemoglobin levels in mice treated with clodronate-liposome and subjected to repeated stress compared with repeatedly stressed mice, suggesting that macrophage depletion markedly worsens anemia under conditions of repeated stress. In addition, both psychological stress and macrophage depletion have been shown to increase hepcidin production [34,35], resulting in significant reductions in plasma iron levels. The clodronate-induced increase in hepcidin production may additionally inhibit dietary iron absorption, thus further worsening low plasma iron levels during repeated restraint stress [7,34]. Significantly decreased hemoglobin concentrations in clodronate-treated and repeatedly stressed mice, as compared with mice that received only clodronate treatment, provide additional support for this hypothesis. However, further research is needed to address this issue and unravel the mechanisms of mutual crosstalk between iron and erythropoiesis under conditions of repeated stress.

In the bone marrow and spleen, erythropoiesis occurs within erythroblastic islands, the distinctive niches in which immature erythroid cells proliferate, differentiate, and enucleate [36]. The erythroid progenitors, BFU-E and CFU-E, characterized by their adaptive ability to self-renew and differentiate, play a critical role in erythropoiesis [37]. Therefore, the regulation of their proliferation is a complex process driven by the cooperative action of different signals, including stem cell factor (SCF) and erythropoietin (EPO) [38]. Under conditions of increased erythropoietic demand, the additional signals, such as BMP4, glucocorticoids, and growth differentiation factor 15 (GDF15), act synergistically with SCF and EPO to provide extensive expansion of erythroid progenitors [3,21,39]. Considering that the muscarinic acetylcholine receptor type-4 negatively regulates BFU-E self-renewal [40], as well as that the restraint stress decreases both the expression and muscarinic receptor binding sites at the periphery [41,42], the extensive expansion of BFU-E cells in the bone marrow and spleen of repeatedly stressed mice may also be a result of decreased acetylcholine signaling. The macrophages constitute a central component of the erythropoietic niche, and their depletion under steady-state conditions did not change the number of BFU-E and CFU-E cells in either the bone marrow or spleen. However, treatment with clodronate-liposomes completely prevented repeated stressed-induced increases in both bone marrow and splenic BFU-E and CFU-E cells, suggesting that macrophages represent the key regulators and main source of locally produced signals for the enhanced proliferation of erythroid progenitors during repeated psychological stress. Similarly, chemical ablation of macrophages fully diminished the increased proliferation of splenic BFU-E cells in the stress erythropoiesis model of polycythemia vera [43] as well as in tumor-induced stress erythropoiesis [44].

During stress erythropoiesis, further differentiation of increased CFU-E output leads to proerythroblasts, which sequentially develop into erythroblasts and finally to mature RBC. Apart from transferrin receptor-CD71, murine erythroid cells at different erythroblast stages also have the Ter119 antigen that is expressed from early proerythroblast to mature erythrocyte [45]. Using the CD71/Ter119 profile of bone marrow and spleen cells, we demonstrated that the depletion of macrophages slightly reduced the percentage of CD71+Ter119+ cells in the bone marrow and did not alter their number in the spleen at steady-state, but it entirely abolished stress-induced increases in the percentage of CD71+Ter119+ within both the bone marrow and spleen. Consistent with these findings, Ramos et al. [33] showed a limited contribution of macrophages to steady-state erythropoiesis but a markedly impaired response of clodronate-treated mice to stress erythropoiesis in a mouse model of phlebotomy-induced anemia. Thus, compared with phlebotomized controls, clodronate-treated phlebotomized mice exhibited a significant reduction in the percentage of CD71+Ter119+ cells in the bone marrow and the spleen. Under conditions of repeated restraint stress, we showed a significantly increased number of more mature CD71−Ter119+ cells in the bone marrow and spleen. Treatment with clodronate-liposomes reversed this effect of stress in the bone marrow, demonstrating the critical role of macrophages in an enhanced bone marrow erythropoiesis during repeated psychological stress. Although slightly reduced, the number of CD71−Ter119+ cells in the spleen of clodronate-treated and stressed mice was not significantly altered, compared with the stressed and clodronate-untreated mice, suggesting that in addition to macrophages, the other splenic cells also contribute to the signals for erythroid cell differentiation in response to stress. Accordingly, EPO is the principal regulator of erythroid cell differentiation [46], and macrophages are an in situ source of EPO for erythroblasts in the bone marrow [47]. However, in the spleen, EPO can also be locally produced by other cells, such as endothelial cells [48] and perivascular stromal cells [49], therefore contributing to the increased number of splenic CD71−Ter119+ cells in repeatedly stressed mice.

Terminal erythroid cell differentiation is characterized by the progressive reductions in cell size, and analysis of Ter119+ cell subpopulations in the bone marrow and spleen revealed that repeated stress increased the percentage of most immature Ter119+ (E1) cells in both the bone marrow and the spleen, whereas it reduced the percentage of more mature Ter119+ (E2) cells in the bone marrow as well as the most mature Ter119+ (E3) cells in the spleen. Erythroid cell proliferation and differentiation are highly coordinated processes that show a remarkable inverse relationship, and some of the key signals for stress erythropoiesis, such as SCF and glucocorticoids, besides inducing an extensive expansion of erythroid progenitors, may also impair terminal erythroid differentiation [50,51], therefore resulting in the reduced percentage of more mature erythroid cells. Thus, Stellacci et al. [50] demonstrated that glucocorticoids inhibit erythroid cell maturation through a membrane-associated pathway that interferes with EPO receptor signaling. Accordingly, our previous results revealed the activation of both glucocorticoid and EPO receptors in the spleen of chronically stressed mice [7] and indicated a physical association between glucocorticoid and EPO receptors during their activation, additionally supporting this notion. Furthermore, the depletion of macrophages completely abolished the stress-induced increase in the percentage of the E1 subpopulation, while it reversed the stress-induced decrease in the percentage of bone marrow E2 cells, as well as the stress-reduced percentage of splenic E3 cells, indicating a substantial contribution of macrophages in obtained stress-induced changes. Accordingly, Ramos et al. [33] demonstrated that macrophages directly promote proliferation and limit enucleation of human primary erythroblasts obtained from patients affected with pathological stress erythropoiesis in β-thalassemia.

Macrophages have been suggested to contribute to HSPC mobilization from the bone marrow during inflammation [12]. It was recently revealed that cholinergic signals via muscarinic receptor type-1 promote the mobilization of hematopoietic stem cells from the bone marrow, indicating that the regulation of HSPC migration emerging from the brain [52]. As the adult spleen contains a low number of immature hematopoietic cells, which cannot fully support an accelerated erythropoiesis during stress conditions, the migration of erythroid progenitors from the bone marrow are strongly needed to expand the splenic erythropoietic niche [53]. Extracellular ATP has been recently recognized as one of the signals that trigger HSPC egress from the bone marrow [54]. Considering that erythroid progenitor cells express P2X7R [55], significantly increased extracellular ATP levels and P2X7R expression in the bone marrow of repeatedly stressed mice may reflect a stress-induced microenvironment, which is supportive for the migration of immature erythroid cells to the spleen. The resemblance between the stress-induced changes in erythroid progenitor cell number and extracellular ATP levels among different experimental groups further supports this hypothesis. Macrophage depletion prevented stress-induced increases in ATP concentrations, suggesting a significant contribution of macrophages to stress-induced extracellular ATP levels in the bone marrow. During stress erythropoiesis, monocytes are recruited to the spleen and generate macrophages that expand the stress erythropoiesis niche [16]. Since monocytes/macrophages express P2X7R and migrate towards increasing ATP concentrations [18], the enhanced levels of extracellular ATP and P2X7R gene expression in the spleen may be a result of the stress-induced supportive microenvironment for monocyte-derived macrophages in an expanding erythropoietic niche. Clodronate treatment revealed an important contribution of resident splenic macrophages to the elevated extracellular ATP levels under conditions of repeated stress. Nevertheless, since the uptake of clodronate-liposomes leads to apoptosis of macrophages, macrophage depletion in the control mice also increased the levels of extracellular ATP. The extracellular ATP are rapidly hydrolyzed by CD39, and the increased specific activity of this enzyme in the bone marrow and spleen of clodronate-treated animals may serve to prevent the toxic and pro-inflammatory effects of persistently high ATP levels [56]. Moreover, the increased specific activity and expression of this enzyme in chronically stressed mice may be protective against an excessive HSPC mobilization from the bone marrow and can contribute to enhanced production of adenosine in the spleen, thus facilitating stress-induced erythropoiesis [57]. Further studies are needed to unravel the molecular mechanisms underlying the contribution of macrophages and extracellular ATP to stress-induced erythropoiesis.

In conclusion, treatment with clodronate-liposome under steady-state conditions induced tissue-specific changes in erythroid cell subpopulations without significantly affecting erythropoiesis in the bone marrow and spleen. In contrast to basal conditions, macrophage depletion completely abolished the stimulatory effect of repeated stress on erythroid progenitors and precursors, extracellular ATP levels, P2X7R expression, and CD39 activity and expression in the bone marrow and spleen, pointing towards a critical role for macrophages in stress-induced changes. Unraveling the mechanisms underlying the contribution of macrophages to stress-induced erythropoiesis may provide new therapeutic strategies for the management of anemia of chronic disease as well as for the suppression of chronic stress erythropoiesis in polycythemia vera.

## 4. Materials and Methods

### 4.1. Animals

Male, 6- to 8-week-old BALB/c mice were obtained from the Breeding Facilities of the Institute for Medical Research, Military Medical Academy, Belgrade. Animals were housed six per cage under conventional conditions (lights on at 06.00 h, lights off at 18.00 h, 21 °C), with a standard laboratory diet and water provided ad libitum.

The experimental protocol was approved by the Ethics Committee of the Institute for Medical Research, University of Belgrade, Serbia (No. O 052-3/21), and the Veterinary Directorate, Ministry of Agriculture, Forestry and Water Management (No. 119-01-511412017-0) according to the National Law on Animal Welfare (Official Gazette of the Republic of Serbia No. 41/09) that is consistent with the guidelines for animal research and principles of the European Convention for the Protection of Vertebrate Animals Used for Experimental and Other Purposes (Official Daily N. L 358/1-358/6, 18 December 1986) and the Directive on the Protection of Animals Used for Scientific Purposes (Directive 2010/63/EU of the European Parliament and of the Council, 22 September 2010).

### 4.2. Experimental Procedure

After an acclimation period of one week, the mice were randomly assigned to the following weight-matched groups: (1) R, restraint group exposed to daily restraint stress; (2) CLOD + R group, received clodronate-liposomes (i.p. 200 μL/20 g body weight) and subjected to daily restraint; (3) CLOD group, treated with clodronate liposomes only; (4) CTRL + R, received control liposomes (i.p. 200 μL/20 g body weight) and subjected to daily restraint; and (5) control, untreated group.

For the restraint stress procedure, mice were individually placed in 50 mL conical centrifuge tubes with multiple ventilation holes, for 2 h per day. Restrained mice were maintained horizontally in their home cages during the restraint sessions and released into the same cage thereafter. The animals were subjected to randomly timed (between 07.00 and 11.00 h) restraint for 7 consecutive days. Intraperitoneal injection of clodronate-liposomes (clodronate liposomes and control liposomes (PBS), Liposoma BV, The Netherlands, SKU: CP 005 005) were used to deplete resident macrophages from the bone marrow and spleen two days prior to the first restraint stress procedure, as well as newly recruited macrophages, every third day for the duration of the experiment. All mice were euthanized by cervical dislocation, femurs, tibias and spleen were harvested under sterile conditions, and single cell suspensions were obtained for further analysis.

### 4.3. Hematologic Parameters

Mouse whole blood was collected into a tube containing EDTA, and RBC counts were made using a hemocytometer. Hemoglobin was analyzed with the cyanmethemoglobin method using Drabkin’s solution (0.1% sodium bicarbonate, 0.005% potassium cyanide, and 0.02% potassium ferricyanide). Values were determined spectrophotometrically at 540 nm and calculated relative to a standard curve. The hematocrit was calculated after brief centrifugation of the blood samples in heparinized microcapillary tubes.

### 4.4. Colony Assays

Single-cell suspensions of bone marrow were prepared by flushing the cells out from the femur and tibia using a syringe and a 21-gauge needle. After harvesting under sterile conditions, the spleens were passed through a wire mash and monodispersed in Dulbecco’s modified Eagle’s medium (DMEM-HPA, Capricorn Scientific, Germany) supplemented with 5% fetal calf serum (FBS, Biowes South America No. S1810). Then, 2 × 10^5^/mL bone marrow cells and 4 × 10^5^/mL splenocytes were separately plated in methylcellulose media (StemCell Technologies, Vancouver, BC, Canada) containing either 3 U/mL EPO (MethoCult M3334) or 3 U/mL EPO supplemented with 50 ng/mL SCF, 10 ng/mL IL-3, and 10 ng/mL IL-6 (MethoCult GF M3434). The cells were plated in duplicate in 35 mm tissue culture dishes (Sarstedt, Germany) and incubated at 37 °C in a humidified atmosphere containing 5% CO_2_. According to the manufacturer’s instructions, burst-forming units-erythroid (BFU-E) were enumerated following the incubation period of 7 days in MethoCult GF M3434 medium, whereas colony-forming unit-erythroid (CFU-E)-derived colonies were scored after 2 days of culture in MethoCult M3334 using an inverted microscope.

### 4.5. Flow Cytometry

Freshly isolated splenocytes and bone marrow cells were monodispersed in DMEM supplemented with 5% FCS. Thereafter, the cells were washed in PBS containing 1 mM ethylene diamine tetra acetic acid (EDTA) and immunostained for 45 min at 4 °C in PBS/0.2% bovine serum albumin with phycoerythrin (PE)-conjugated anti-mouse Ter119 (1:200, Cat. No. 553673, BD Pharmingen, San Jose, CA, USA) and PE/Cy7-conjugated anti-mouse CD71 antibody (1:200, Cat. No. 113811, BioLegend, San Diego, CA, USA). Following a subsequent washing, the cells were resuspended in 1 mL PBS and analyzed using a CyFlow SL flow cytometer (Partec, Münster, Germany).

### 4.6. RNA Extraction and Real-Time PCR

Total RNA was extracted from homogenized spleen samples using standard methods and TRIzol reagent (Invitrogen, Carlsbad, CA, USA, No. 15596026) according to the manufacturer’s recommendations. The RNA was assessed for quantity and integrity using the ImplenNanoPhotometer P330 (Implen GmbH, München, Germany). Complementary DNA was produced using High Capacity cDNA Reverse Transcription Kit (Applied Biosystems, Waltham, MA, USA, Cat No. 4374966). Real-time quantitative PCR was performed on a Mic qPCR cycler (Bio Molecular Systems, Queensland, Australia) using the Fast Green Kit (Applied Biosystems, Foster City, CA, USA) and the following oligonucleotide primers: P2X7R (forward): 5′-TGCAGCTGGAACGATGTCTT-3′; P2X7R (reverse): 5′-GACGGTGCCATAATTCGTGC-3′; CD39 (forward): 5′-TACCACCCCATCTGGTCATT-3′; CD39 (reverse); 5′-GGACGTTTTGTTTGGTTGGT-3; β actin (forward): 5′-GCTGTATTCCCCTCCATCGT-3′; β actin (reverse): 5′-TTCTCCATGTCGTCCCAGTT-3′. PCR reactions were started with an initial denaturation at 95 °C for 20 s, followed by 40 cycles each consisting of denaturation at 95 °C for 3 s, and annealing/extension at 60 °C for 30 s. A melting curve analysis was performed after amplification was completed. The relative expression of P2X7R and CD39 genes was quantified using the ΔΔCt method normalized to the expression of the β-actin gene.

### 4.7. Extracellular ATP Measurement

Single-cell suspensions of bone marrow and spleen samples were obtained from each mouse and 1 × 10^6^ cells were plated in duplicate. Four hours after seeding cells from the bone marrow and spleen, the medium was collected and centrifuged at 310× *g* for 8 min. The supernatants were used to quantify the extracellular ATP by bioluminescence assay (CellTiter-Glo, Promega, Madison, WI, USA, Cat. No. G7570) according to the manufacturer’s instructions.

### 4.8. CD39 Activity

Cells in the pellet were washed twice with phosphate-free reaction medium (120 mM NaCl, 5 mM KCl, 2 mM MgCl_2_x6H_2_O, 10 mM glucose, 20 mM HEPES, pH 7.4) and pre-incubated at 37 °C for 5 min. To determine CD39 activity, 200 µL of the reaction medium containing 5 × 10^5^ cells was incubated in the presence of 0.5 mM ATP for 10 min. The reaction was stopped by placing the tubes on ice and centrifuging at 4 °C for 10 min (310× *g*). The supernatant was collected and used for the colorimetric assay with malachite green [58]. Two aliquots of the supernatant (80 µL) of each reaction sample were placed in a 96-well plate and mixed with 20 µL of the malachite green reagent. The plate was incubated for 30 min at room temperature with shaking. Absorbance at 650 nm was determined using the Wallac Victor 1420 Multilabel Counter (Perkin-Elmer Life Science, Waltham, MA, USA). Inorganic phosphate (Pi) was quantified using KH_2_PO_4_ as a standard (concentration range: 4–40 μM). To correct for non-enzymatic ATP hydrolysis, Pi was measured in the reaction medium without cells and subtracted from the total Pi released during incubation.

The remaining cell pellets were dried and frozen for protein determination by the method of Markwell using BSA as a standard [59]. The enzyme activities were expressed as nmol Pi min/mg/protein.

### 4.9. Western Blot

For protein analysis, the spleens were homogenized in chilled RIPA buffer (50 mM Tris-HCl, pH 7.6, 150 mM sodium chloride, 1%Triton x-100, 1% sodium deoxycholate, 0.1% sodium dodecyl sulphate, 2 mM EDTA, 1 mM DTT, 50 mM sodium fluoride) with protease inhibitor cocktail (Pierce, Thermo Fisher Scientific, Rockford, IL, USA). The isolated bone marrow cells were washed with PBS and lysed in chilled RIPA buffer supplemented with protease inhibitors. The cell lysates were cleared by centrifugation (10,000× *g* for 10 min at 4 °C) and stored at −70 °C until analysis. The protein concentrations were determined by the BCA Protein Assay Kit (Pierce, Thermo Fisher Scientific, Rockford, IL, USA). For Western blotting, equal amounts of protein from each sample were loaded and resolved on SDS-PAGE (SE 260, Mini Vertical Electrophoresis Unit, Hoefer Inc., San Francisco, CA, USA), and transferred to a nitrocellulose membrane (Serva Electrophoresis GmbH, Heidelberg, Germany) using a semi-dry blotting system (TE77X Semidry Blotting Systems, Hoefer Inc., San Francisco, CA, USA). The membranes were probed with primary antibody to F4/80 [60,61] (#70076, Cell signaling, Danvers, MA, USA) and actin-specific antibody (MAB8929, R&D Systems, Minneapolis, MN, USA). Peroxidase-conjugated goat anti-rabbit immunoglobulin (R&D Systems, Minneapolis, MN, USA.) and goat anti-mouse immunoglobulin (Sigma-Aldrich, Merck, Darmstadt, Germany) were used as secondary antibodies. Actin was used as a loading control. The Western blots were developed using the enhanced chemiluminescence reagent system (Serva Electrophoresis GmbH, Heidelberg, Germany) according to the manufacturer’s instructions. Chemiluminescent visualization of images was performed using ChemiDoc Imaging System (Bio-Rad, Hercules, CA, USA).

### 4.10. Statistical Analysis

Data are expressed as mean ± SEM of 6–8 mice per group. Differences across treatment groups were tested for statistical significance using the analysis of variance (ANOVA). Following the ANOVA, post hoc comparisons using Bonferroni-corrected *t* test, Fisher’s least significant difference test, or Games–Howell were performed as appropriate. Quantitative RT-PCR analysis was carried out with the Relative Expression Software Tool (REST, https://www.gene-quantification.de/rest-2009.html, accessed on 8 July 2023) 2009 using the pairwise fixed randomization test [62]. Significance was determined at the *p* < 0.05 level.

## Figures and Tables

**Table 1:** Hematological parameters.

	C	R	CLOD	CLOD + R	CTRL + R
RBC (10^12^/L)	5.52 ± 0.16	5.35 ± 0.18	4.54 ± 0.12	3.95 ± 0.06 *^(a)^***^(b)^	5.18 ± 0.19
Hb (g/L)	165.34 ± 1.95	130.57 ± 2.68 ***^(a)^	140.26 ± 3.14 ***^(a)^	100.68 ± 1.68 ***^(a)^***^(b)^	132.94 ± 2.48
Hct (%)	45.67 ± 1.17	43.75 ± 1.71	40.67 ± 0.56 **^(a)^	39.12 ± 0.64 **^(a)^	42.83 ± 1.3
MCV (fl)	83.78 ± 3.84	86.26 ± 1.79	84.66 ± 2.49	92.51 ± 4.45	85.65 ± 2.26
MCH (pg)	30.06 ± 0.88	24.41 ± 0.72 **^(a)^	27.26 ± 0.9	24.72 ± 0.73 **^(a)^	25.84 ± 1.14 *^(a)^
MCHC (g/L)	363.18 ± 9.54	301.56 ± 13.03 **^(a)^	344.94 ± 6.6	278.20 ± 5.42 ***^(a)^	311.58 ± 9.87 *^(a)^
RDW (%)	11.36 ± 0.54	14.01 ± 0.28 *^(a)^	11.97 ± 0.38	13.46 ± 0.87	13.78 ± 0.6

Differences between groups were assessed by ANOVA and post hoc Bonferroni-corrected *t*-test or Games–Howell test. *** *p* < 0.001; ** *p* < 0.01; * *p* < 0.05; ^(a)^ compared with C (control) group; ^(b)^ compared with R (restraint) group. Data are expressed as mean ± SEM (*n* = 6–8/group). Abbreviations: RCB—red blood cells; Hb—hemoglobin; Hct—hematocrit; MCV—mean corpuscular volume; MCH—mean corpuscular hemoglobin; MCHC—mean corpuscular hemoglobin concentration; RDW—red cell distribution width.

## Data Availability

The data presented in this study are available from the corresponding author upon reasonable request.

## Supplementary Materials

The following supporting information can be downloaded at: https://www.mdpi.com/article/10.3390/ijms241411373/s1.
