# Supplementary figures and images for: Macrophages Provide Essential Support for Erythropoiesis, and Extracellular ATP Contributes to a Erythropoiesis-Supportive Microenvironment during Repeated Psychological Stress
Source: Int J Mol Sci. 2023 Jul 12;24(14):11373. doi: 10.3390/ijms241411373 (PMC10379406; doi:10.3390/ijms241411373)

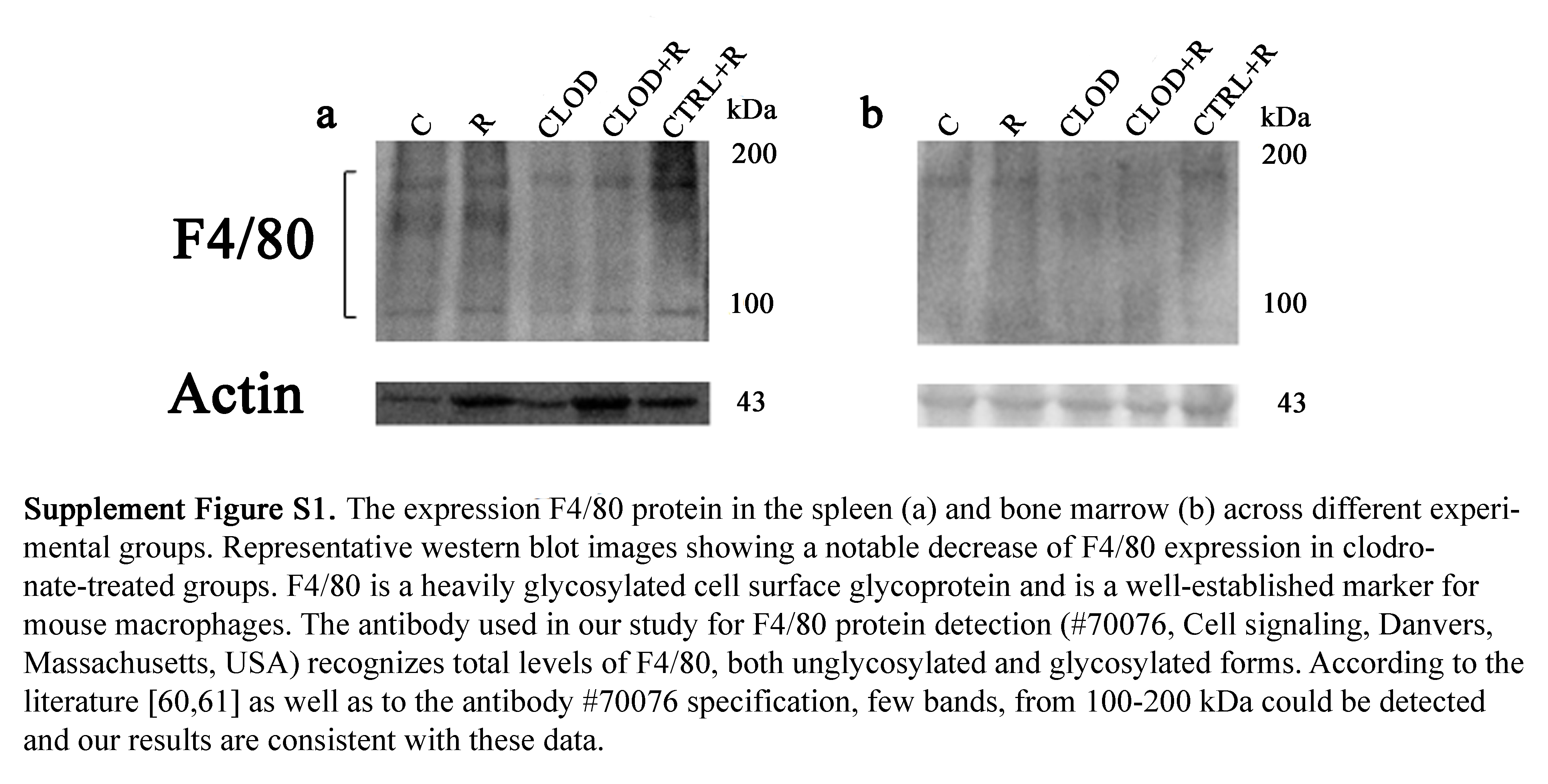

Supplement: Supplementary file 1 [file ijms-24-11373-s001.zip › ijms-2451513-supplementary/Supplement Figure S1.tif]

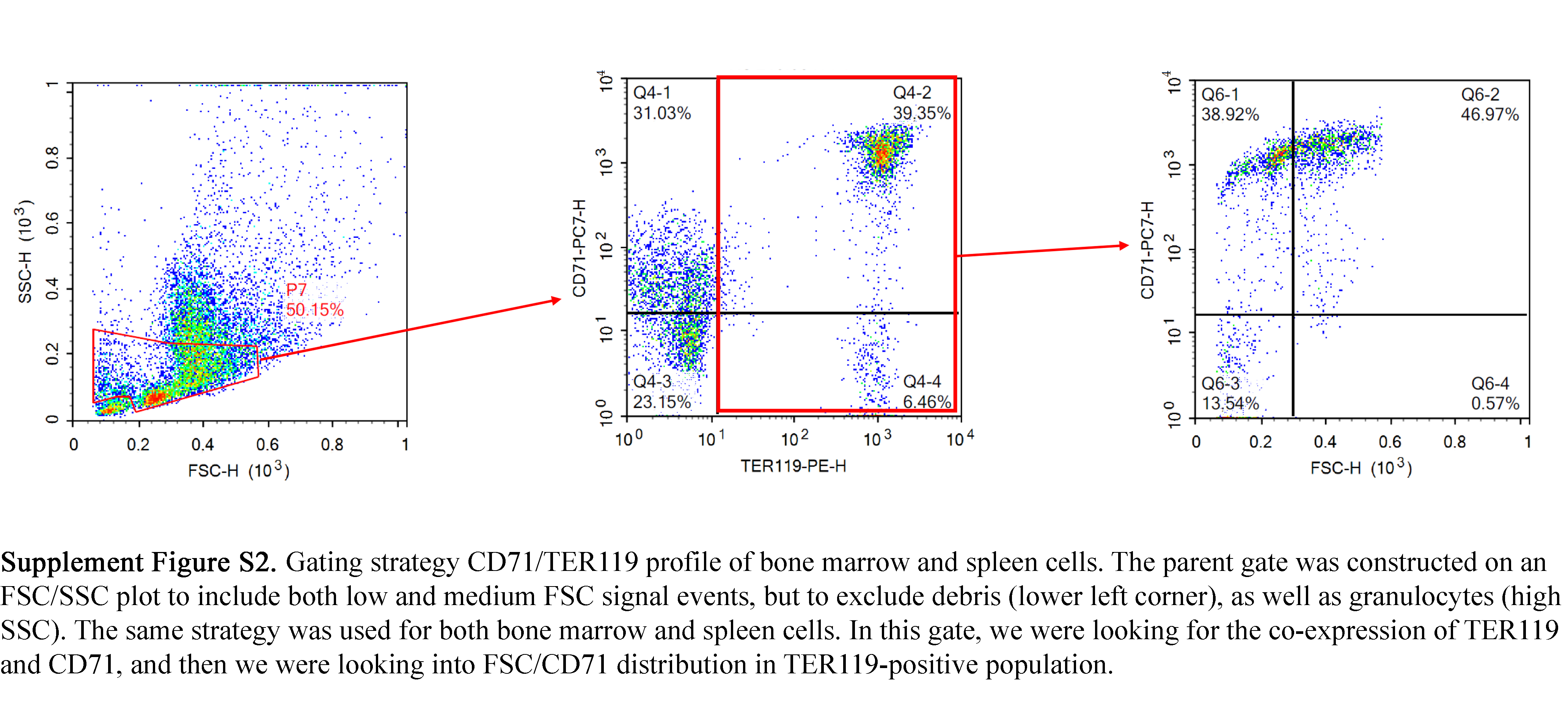

Supplement: Supplementary file 1 [file ijms-24-11373-s001.zip › ijms-2451513-supplementary/Supplement Figure S2.tif]
